# Phenotypic and Enzymatic Comparative Analysis of the KPC Variants, KPC-2 and Its Recently Discovered Variant KPC-15

**DOI:** 10.1371/journal.pone.0111491

**Published:** 2014-10-31

**Authors:** Dongguo Wang, Jiayu Chen, Linjun Yang, Yonghua Mou, Yijun Yang

**Affiliations:** 1 Department of Clinical Lab Medicine, Taizhou Municipal Hospital affiliated with Taizhou University and the Institute of Molecular Diagnostics of Taizhou University, Zhejiang, China; 2 Department of Lab Medicine, Medical School and the Institute of Molecular Diagnostics of Taizhou University, Zhejiang, China; 3 Department of Thyroid Breast Surgery, Taizhou Municipal Hospital affiliated with Taizhou University, Zhejiang, China; 4 Department of Hepatobiliary Surgery, Taizhou Municipal Hospital affiliated with Taizhou University, Zhejiang, China; 5 Department of Quality Control, Taizhou Municipal Hospital affiliated with Taizhou University, Zhejiang, China; Universidad de Granada, Spain

## Abstract

Sixteen different variants (KPC-2 to KPC-17) in the KPC family have been reported, and most current studies are focusing on KPC-2 and KPC-3. The KPC-15 variant, which isolated from *Klebsiella pneumoniae* in a Chinese hospital, was a recently discovered KPC enzyme. To compare the characteristics of KPC-15 and KPC-2, the variants were determined by susceptibility testing, PCR amplification and sequencing, and study of kinetic parameters. The strain harboring the KPC-15 showed resistance to 18 conventional antimicrobial agents, especially to cabapenem antibiotics, and the strain involving the KPC-2 also indicated resistance to cabapenem antibiotics, but both strains were susceptible to polymyxin B and colistin. The conjugation experiments showed that the changes of MIC values to the antibiotics were due to the transferred plasmids. The differences of amino acids were characterised at sites of 119 leucine and 146 lysine with KPC-15 and KPC-2. The minimum evolution tree indicated the KPC alleles evolution, and showed that the KPC-15 appeared to be homogenous with KPC-4 closely. Steady-state kinetic parameters showed the catalytic efficiency of KPC-15 was higher than that of KPC-2 for all tested antibiotics in this study. The catalytic efficiency of KPC-15 caused resistance to β-lactam antibiotics was higher than that of KPC-2. Meanwhile, an evolutionary transformation changed KPC from an efficient carbapenemase to its variants (KPC-15) with better ceftazidimase catalytic efficiency, and the old antibiotics polymyxin B and colistin might play a role in the therapy for multi-resistant strains.

## Introduction

The recent emergence and spread of acquired carbapenem-resistance in gram-negative bacteria, which resulting in more clinical failures in infection control, have been increasingly reported. Carbapenem-resistant *Enterobacteriaceae* are usually resistant not only to β-lactam antibiotics but also to most other classes of antimicrobial agents [1].

Resistance to carbapenems in gram-negative bacteria is frequently observed due to the high expression of AmpC β-lactamases, which are associated with the loss of outer membrane proteins [2], the increased expression of efflux pumps systems [3], and with changes in the affinity of penicillin-binding proteins (PBPs) for carbapenems [4]. Carbapenem resistance can primarily be mediated by carbapenem-hydrolysing enzymes, such as metallo-β-lactamases (MBLs), Guiana extended-spectrum (GES) and *Klebsiella pneumoniae* carbapenemases (KPCs) [5]–[7].

KPC was first discovered in a *K. pneumoniae* clinical isolate collected from North Carolina in 2001 [8]. Thereafter, an amino acid variant of KPC-1, which is termed KPC-2, is discovered in *Klebsiella* spp. [9], [10] and *Salmonella enterica* [11]. However, a recent correction of the *bla*_KPC-1_ sequence have indicated that the sequences *of bla*_KPC-1_ and *bla*_KPC-2_ are identical, so, KPC-1 and KPC-2 are the same enzymes [8]. Mutations in the *bla*_KPC-2_ gene have resulted in two variants, *bla*_KPC-3_ [12] and *bla*_KPC-4_ [13]. Since then, KPC have been detected in several members of *Enterobacteriaceae* and discovered in American, Asian, European and Near East regions [7], [14]–[20].

Currently, sixteen different variants (KPC-2 to KPC-17) in the KPC family have been reported, and most current studies are focusing on KPC-2 and KPC-3 [21]–[23]. In the study, we characterised phenotypic and enzymatic comparative analysis for the KPC-2 enzyme and novel KPC-15 variant which was discovered recently by us [24].

## Materials and Methods

### The ethics committee approval

This study was approved by the the Ethics Committee of Municipal Hospital of Taizhou University, and the written informed consents obtained from each of the participants. The right of research objects was protected, and we agreed this study was conducted in our hospital.

### Bacterial strains

The two strains of *K. pneumoniae*, which were numbered Kp1769 and Kp1241, were isolated from samples of sputum and blood, respectively. The strain Kp1241 was isolated from the hepatobiliary unit in June 2012, and the strain Kp1769 was isolated from the ICU in September 2011 in respiration unit in the Taizhou Municipal Hospital of Taizhou University. The strain Kp1769, which was confirmed involving the *bla*_KPC-2_ gene, was used to compare the characteristics of the kinetic parameters with the recently discovered *bla*_KPC-15_ variant in this study. These two strains were assigned to *K. pneumoniae* using a Vitek GNI^+^ card (bioMérieux, France), and species identification was confirmed by a sequence analysis of the 16 S-23 S rRNA (Midi Labs, USA).

### Antimicrobial susceptibility testing and conjugation experiments

The MICs of 20 antimicrobial agents, including imipenem, meropenem, ertapenem, ampicillin, aztreonam, cefazolin, cefepime, ceftazidime, ceftriaxone, ciprofloxacin, levofloxacin, amikacin, tobramycin, gentamicin, cephalosporin, ampicillin/sulbactam, piperacillin/tazobactam, cefoperazone/sulbactam, polymyxin B and colistin in the strains of *E. coli* J53Az^R^, Kp1241, Kp1769 and transconjugants, were determined using the Micro- Scan (USA) broth dilution method with plates. *Escherichia coli* ATCC 25922 and *Pseudomonas aeruginosa* ATCC 27853 were used as the controls. The results were interpreted according to CLSI (2012) [25].

According to a previously reported protocol [26], conjugation experiments were performed in lysogeny broth (LB) with the strain *E. coli* J53Az^R^ (resistance to sodium azide) as the recipient and with the strains Kp1241 and Kp1769 as the donor strains. Donor and recipient cells in logarithmic phase (0.5 mL of each) were added to 4 mL of fresh LB, which was followed by incubation at 35°C for 18–24 h without shaking. The transconjugants were selected on trypticase soy agar (TSA) plates containing sodium azide (0.3 g/L) and imipenem (0.02 g/L) following incubation for 18–24 h at 35°C.

### PCR amplification and sequencing

PCR was performed for the amplification and sequencing of pre-*bla*_KPC-15_, the *bla*_KPC-15_ variant, and its surrounding genes (*bla*_TEM_, *tnpA* and *tnpR*) in *K. pneumonae* using the primers shown in Table 1. PCRs were also performed for the amplification of the transformant conjugated with *E. coli* J53Az^R^. The primers were synthesised by Sangon Biological Technology (Shanghai, China). The PCR conditions for pre-*bla*_KPC-15_ and the *bla*_KPC-15_ variant were as follows: 3 min at 94°C and 30 cycles of 1 min at 94°C, 1 min at 52°C, and 1 min at 72°C, followed by an elongation step for 10 min at 72°C, which produced a band of ca. 1,431 bp for *bla*_KPC-15_, encompassing the entire KPC coding region. The same PCR conditions were used to amplify *bla*_TEM_, *tnpA* (IS*Kpn8* and IS*Kpn6-*like transposases) and *tnpR* genes. The primers of tnpA_1_, tnpA_2_ and tnpA_3_ were used to amplify the IS*Kpn8* transposon, and the tnpA primer was used to amplify the IS*Kpn6-*like transposon. The positive PCR products were also sequenced by Sangon Biological Technology (Shanghai, China). Then, the sequencing results were assembled using the contigExpress Program software and the sequences were obtained for the genetic environment of the *bla*_KPC-15_ variant from the strain Kp1241.

**Table 1:** Sequences of primers utilised to determine the *bla*_KPC_ genetic environment in this study.

Primer	Sequence (5′-3′)	Productsize (bp)	reference
pre-KPC-15	For, ACTGGATGGAGGCGGATAA	700	The study (based on GenBank accession no. AY395881)
	Rev, CACAGCGGCAGCAAGAAA		
pre-KPC-15 (sequencing)	For, TAAGCCCTCCCGTATCGTAG		The study (based on GenBank accession no. AY395881)
	Rev, TATCCGCCTCCATCCAGT		
KPC-15	For, CGAGCAACTATGGATGAACG	1,431	The study (based on GenBank accession no. AY395881)
	Rev, GTATCTGTGAGGGCGAAGG		
KPC-15 (sequencing)	For, CGGAACCATTCGCTAAACTCG		The study (based on GenBank accession no. AY395881)
	Rev, CGCCAACTCCTTCAGCAACA		
tnpA_1_	For, ATCCGCATCGGGAAAGCC	1144	The study (based on GenBank accession no. JX500679)
	Rev, CTGCTCGTCGGCAAAGGA		
tnpA_2_	For, GCCAAGACACTGCTGCCTAA	1082	The study (based on GenBank accession no. JX500679)
	Rev, TTGACGGAAGCGAAACACG		
tnpA_3_	For, CAAGTTTCTGTCGGCCTTCA	1434	The study (based on GenBank accession no. JX500679)
	Rev, CACGCCTTTGCTCCTGGGT		
tnpA	For, ATACGCCATTCGCCTCAG	1320	The study (based on GenBank accession no. JX500679)
	Rev, GTCGGCAAGGTGGTCTCA		
tnpR	For, TCTTCGCAACACGCACCA	1834	The study (based on GenBank accession no. JX500680)
	Rev, ACCTCGCCGTGGAAATAG		
TEM	For, CTGTCTATTTCGTTCATCC	1061	The study (based on GenBank accession no. X64523)
	Rev, CTCAGTATTGCCCGCTCC		

Plasmids were isolated from the strain Kp1241 using an Axygen kit (USA) in accordance with the manufacturer’s instructions. Plasmids were then separated by electrophoresis through a 0.6% agarose gel in the presence of 1× TBE buffer at a constant voltage of 90 V for 90 min. These plasmid DNA fragments served as samples for the amplification of *bla*_KPC-15_ gene by PCR, where the initial position of the *bla*_KPC-15_ gene plasmid was determined. The estimated sizes of plasmid DNAs were determined according to reference [26]. Based on these data, we mapped the genetic sequence of the *bla*_KPC-15_ upstream and downstream regions and determinded the start sites of transcription and the promoter regions of relevant genes.

### β-lactamase preparation and the kinetic parameters for a comparison of KPC-15 and KPC-2

Cultures of *K. pneumoniae* harboring *bla*_KPC-15_ and *bla*_KPC-2_ were grown overnight at 37°C in 100 ml of trypticase soy broth with amoxicillin (100 mg/mL). Bacterial suspensions were disrupted by sonication (20 s at 20 KHz×4 times) and centrifuged (30 min, 20,000 g, 4°C). The supernatants, which contained the crude enzyme extracts, were collected. Then, the crude enzyme extracts were mixed with 50 mM Tris-Cl, pH 7.4, with 1 mM magnesium sulphate, and lysed with 40 mg/L lysozyme. Next, 1.0 U/ml benzonase nuclease was added to digest nucleic acids, and a 2.0 mM concentration of EDTA was added to complete the periplasmic fractionation. The lysed cells were centrifuged at 12,000 rpm for 10 min to remove the cellular debris. The supernatant was further enriched and purified for β-lactamase by using preparative isoelectric focusing as described previously [27], [28]. The β-lactamase purification step was performed in accordance with reference [29].

Steady-state kinetic parameters were determined using a diode array spectrophotometer (Agilent, USA). Each assay was performed with 10 mM PBS, pH 7.4, at 25°C. In all assays, the enzyme was maintained at 10 nM, whereas substrate concentrations were varied from 50 to 200 µM. Kinetic assays were performed under steady-state conditions to determine the kms and kcats of the antimicrobial agents: imipenem, meropenem, ceftazidime, cefotaxime, cefepime, aztreonam and nitrocefin for the enzyme according to a previously established model represented in the kinetic parameter equation [30], [31] and in reference [29]. Concentrations varied from 25 to 250 µM for imipenem; 50 to 250 µM for meropenem; 150 to 500 µM for ceftazidime; 100 to 500 µM for cefotaxime; 250 µM to 1.5 mM for cefepime; and 50 mM to 500 mM for aztreonam. A final concentration of 100 µM nitrocefin was used as the reporter substrate.

## Results

### Antimicrobial susceptibility testing and conjugation experiment

The MICs of 20 antimicrobial agents for the strains Kp1241 and Kp1769 were depicted in Table 2. The results suggested that the strain Kp1241 was resistant to 18 antimicrobial agents, and particularly exhibited high MIC values for imipenem, meropenem, and ertapenem at 16, 16, and 32 µg/mL, respectively; however, the strains Kp1769 showed MIC values for imipenem, meropenem, and ertapenem at 2, 2, and 4 µg/mL, respectively. And the strain Kp1241 remained susceptible to polymyxin B and colistin, so it was as the strain Kp1769. By the conjugation experiments, the transconjugant of strain Kp1241 exhibited a phenotype of resistance to imipenem, meropenem, ertapenem, cephalosporin, aminoglycosides, ampicillin/sulbactam, piperacillin/tazobactam and cefoperazone/sulbactam, but, the transconjugant of strain Kp1769 indicated intermedium values to imipenem, meropenem, and ertapenem. Hence, the results of conjugation experiments showed that the changes of MIC values of the antibiotics were due to the transferred plasmids.

**Table 2:** The susceptibility for strains of *E. coli* J53Az^R^, Kp1241, Kp1769, and transconjugants (J53Az^R^-Kp1241 and J53Az^R^-Kp1769).

Antimicrobial agent	MIC (µg/mL)				
	*E.coli* J53Az^R^	Kp1241	Kp1769	J53Az^R^-Kp1241	J53Az^R^-Kp1769
Ciprofloxacin	0.0025	16	0.25	4	0.0625
Levofloxacin	0.0025	16	0.25	8	0.625
Amikacin	0.25	128	2	32	1
Tobramycin	0.025	64	1	32	0.5
Gentamicin	0.25	32	1	16	0.5
Ampicillin	0.050	64	128	32	32
Aztreonam	0.50	128	2	32	1
Cefotetan	0.0125	128	64	32	16
Cefazolin	0.50	128	64	64	16
Cefepime	0.0125	128	1	32	0.625
Ceftazidime	0.050	16	2	8	1
Ceftriaxone	0.025	128	16	32	4
Imipenem	0.25	16	2	8	1
Meropenem	0.25	16	2	8	1
Ertapenem	0.50	32	4	16	2
Ampicillin/Sulbactam	0.050	128	64	32	16
Piperacillin/Tazobactam	0.025	256	16	64	4
Cefoperazone/Sulbactam	0.0125	128	2	32	0.5
Polymyxin B	0.006	0.5	0.25	0.125	0.0625
Colistin	0.0125	1	0.5	0.25	0.125

Clinical breakpoints of MICs for the antimicrobial agents see the reference [25].

### Amino acid variations, the minimum evolution tree of amino acid sequences and the kinetic parameters for KPC-15 and other KPC enzymes

**Figure 1:**
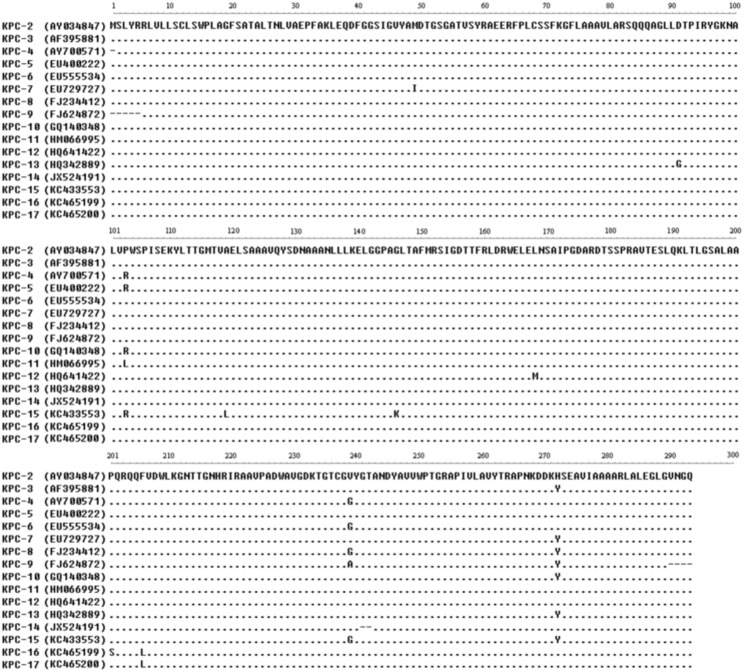
Comparison of amino acid sequence alignments of KPC carbapenemases. KPC-15 was recently identified as a carbapenemase in *Klebsiella pneumonae* from the Taizhou Municipal Hospital of China. The data within parentheses are GenBank accession numbers.

**Figure 2:**
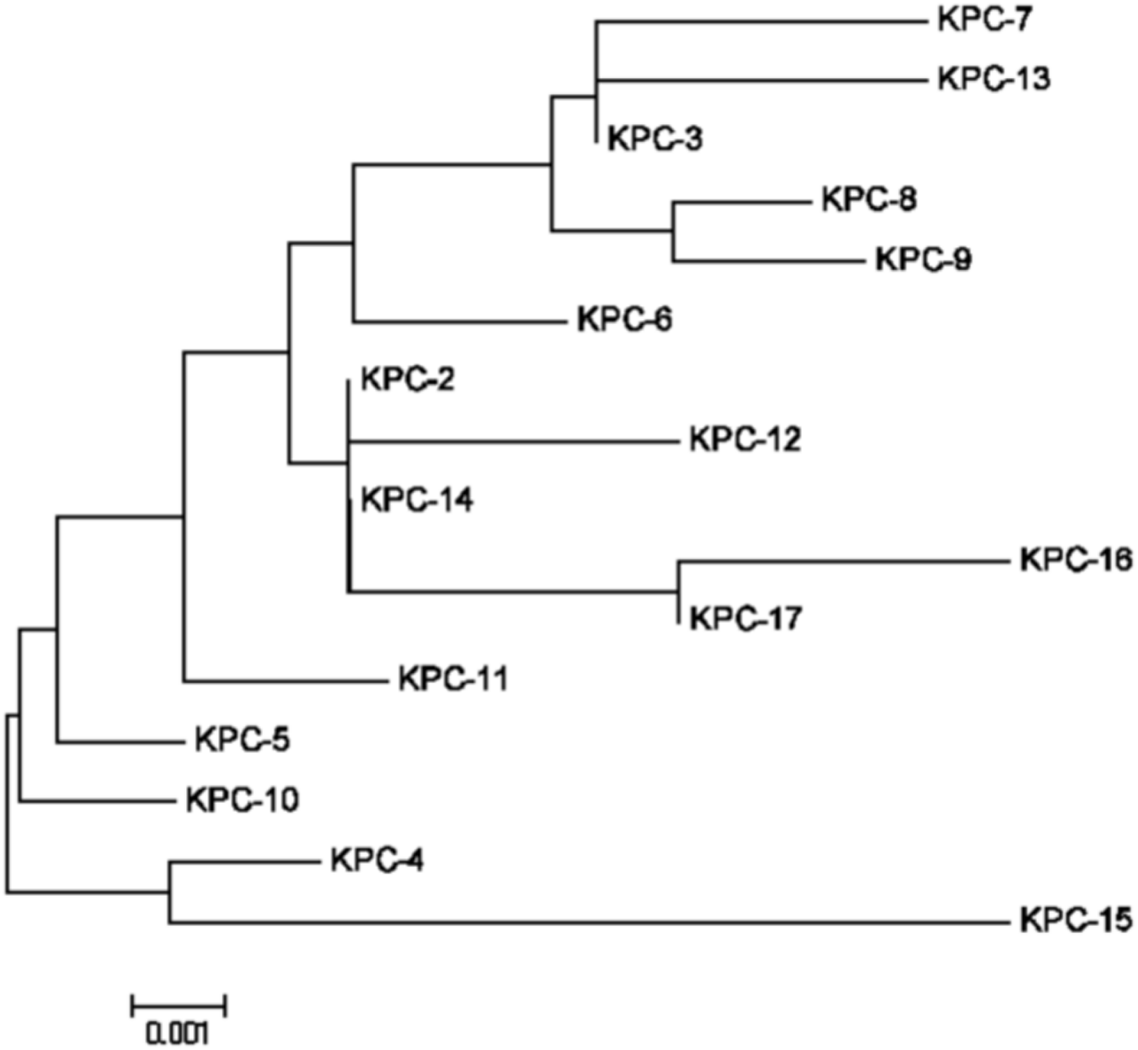
Minimum Evolution tree of amino acid sequences for KPC-2 to KPC-17. KPC-15 carbapenemase was our newly discovered and appeared to be homogenous with KPC-4 closely. The amino acid sequences of KPCs based on the data of Figure 1. This comparison was designed and analysed using MEGA 5.05 software.

Amino acid variations between novel *bla*_KPC-15_ and other *bla*_KPC_ genes were listed in Figure 1, and basing on the data of Figure 1, we drew the minimum evolution tree of amino acid sequences for KPC-2 to KPC-17 (Figure 2) using MEGA 5.05 software. The tree obviously indicated the KPC alleles evolution, and showed that the KPC-15 appeared to be homogenous with KPC-4 closely.

Steady-state kinetic parameters were measured five times and averaged as shown in Table 3. Consistent with the MIC data, the catalytic efficiency (kcat/km ratio) of the KPC-15 enzyme was lowest for ceftazidime, at 0.0142 µM^−1^ s^−1^, and was higher at 5.26-fold compared with that of the KPC-2 enzyme, at 0.0027 µM^−1^ s^−1^. However, the catalytic efficiency of the KPC-15 enzyme was highest for nitrocefin, at 9.2 µM^−1^ s^−1^, and was a little higher at 1.10-fold compared with that of the KPC-2 enzyme, at 8.4 µM^−1 ^s^−1^. The catalytic efficiency of the KPC-15 enzyme was higher at 2.96-fold for imipenem, with a value of 2.81 µM^−1^ s^−1^, compared with that of the KPC-2 enzyme, at 0.95 µM^−1^ s^−1^. Additionally, the catalytic efficiency of the KPC-15 enzyme was also higher at 2.72-fold for meropenem, with a value of 0.68 µM^−1^ s^−1^, compared with that of the KPC-2 enzyme, at 0.25 µM^−1^ s^−1^; the catalytic efficiency of KPC-15 enzyme was higher at 2.19-fold and 2.43-fold for cefotaxime and aztreonam, with values of 0.92 and 0.34 µM^−1^ s^−1^, respectively, compared with those values of the KPC-2 enzyme, at 0.42 and 0.14 µM^−1^ s^−1^, respectively, but higher at 2.01-fold for cefazolin, with a value of 5.62 µM^−1^ s^−1^, compared with that of the KPC-2 enzyme, at 2.8 µM^−1^ s^−1^. The quantitative values of the initial rate versus substrate concentration (v_0_/[s]) for KPC-15 and KPC-2 enzymes listed in Table S1. In general, the catalytic efficiency of KPC-15 was higher than that of KPC-2 for all tested antibiotics in this study (Table 3).

**Table 3:** Kinetic parameters for KPC-15 and KPC-2 enzymes.

Substrate	KPC-15					KPC-2	
	km (µM)	kcat (s^−1^)	kcat/km (µM^−1^s^−1^)	km (µM)	kcat (s^−1^)		kcat/km (µM^−1^s^−1^)
Imipene	69±3	194±2	2.81±0.54	21±2	20±1		0.95±0.65
Meropenem	19.6±2.1	13.4±0.95	0.68±0.73	16.2±3.1	4.1±0.99		0.25±0.061
Ceftazidime	192±8	2.73±0.91	0.0142±0.00074	217±6	0.59±0.83		0.0027±0.00056
Cefotaxime	101±7	93±9	0.92±0.18	124±6	52±6		0.42±0.13
Aztreonam	373±4	126±6	0.34±0.053	389±5	53±2		0.14±0.077
Cefazolin	21±2	118±2	5.62±0.87	16±1	44±2		2.8±1.0
Nitrocefin	5.2±1.1	47.6±3.2	9.2±0.96	6.5±2.4	54.7±4.1		8.4±0.34

### PCR sequencing analysis and genetic organisation

**Figure 3:**
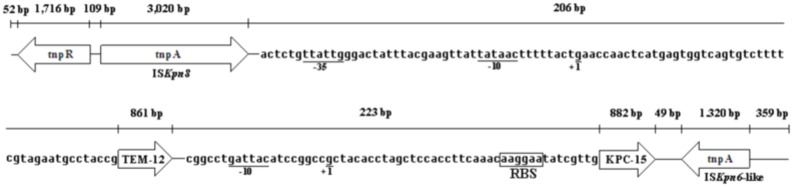
Promoter elements of the *bla*_KPC-15_ and *bla*_TEM_ genes in 8.997 kb-length nucleotide sequence. The sequence provided the upstream and downstream regions of *bla*_KPC-15_ structural genes. The *tnpA* gene, which was upstream of the *bla*_KPC-15_ (3,020 bp) gene, was homologous to a putative IS*Kpn8* transposon, and the downstream region of the *bla*_KPC-15_ gene (1,320 bp) was homologous to a putative IS*Kpn6*-like transposon. The nucleotides upstream of the *bla*_KPC-15_ and *bla*_TEM-12_ gene translational start codons were shown in the box. The putative −10 promoter elements of the *bla*_KPC-15_ gene were shown as *gattaa*, labeled as −10 below, and there were no obvious −35 promoter elements to be discovered in the promoter region. The putative −10 and −35 promoter elements of the *bla*_TEM-12_ gene were shown as *tataac* and *ttattg*, labeled as −10 and −35 below the promoter region. The start sites of transcription were indicated as G by +1 residue. RBS was the abbreviation of the ribosome binding site.

The analysis of the 8,997 bp nucleotide sequence for Kp1241 was shown in Figure 3. There were 5 different genes in the sequence, including the genes *tnpR*, *bla*_TEM-12_ and *bla*_KPC-15_, and the transposons IS*Kpn8* and IS*Kpn6*-like [24]. The sites of the transcriptional promoters for the *bla*_TEM-12_ and *bla*_KPC-15_ genes were indicated as +1 and the −10 and −35 regions were also shown; however, there were no obvious transcriptional promoters in *tnpR*, IS*Kpn8* and IS*Kpn6*-like transposons. Between the *tnpR* gene and the IS*Kpn8* transposon, there was a 109 bp nucleotide interval; the IS*Kpn8* transposon nucleotide sequence was 3,020 bp. Between the IS*Kpn8* transposon and the *bla*_TEM-12_ gene, there was a 206 bp nucleotide interval, which included the site of the transcriptional promoter for the *bla*_TEM-12_ gene, +1 (*g*, start codon), −10 region (*tataac*) and −35 region (*ttattg*). Between the *bla*_TEM-12_ and *bla*_KPC-15_ genes, there was a 223 bp nucleotide interval, which included the site of the transcriptional promoter for the *bla*_KPC-15_ gene, +1 (*g*, start codon), −10 region (*gattac*) and RBS (*aaggaa*, the ribosome binding site), but no obvious −35 region was shown in the *bla*_KPC-15_ gene. Between the *bla*_KPC-15_ gene and the IS*Kpn6*-like transposon, there was a 249 bp nucleotide interval, and the IS*Kpn6*-like transposon had a 1,320 bp nucleotide sequence.

## Discussion

In recent years, many reports have described the presence of KPC β-lactamases (particularly KPC-2), and discovered they can cause resistance to carbapenem or reduce susceptibility to carbapenem in strains of the family of *Enterobacteriaceae* from different areas in China [5], [16], [32]. It seems that KPC enzymes are becoming widely disseminated in China, and plasmids of different sizes may harbor the *bla*_KPC-2_ gene [33]. The genetic environments of the *bla*_KPC_ gene have been characterised in some studies, and various transposon elements seems to be responsible for the rapid spread of *bla*_KPC_ [33]–[35].

In this study, the susceptibility tests indicated that the strain Kp1241 showed resistance to quinolones, aminoglycosides, and the beta-lactam antibiotics, but susceptibility to polymyxin B and colistin with MICs of 0.5 and 1 µg/mL (Table 2), respectively. This result suggested that the old antibiotics, polymyxin B and colistin, might play an important role in the treatment of the strain Kp1241 [36].

Comparing the amino acids with other KPCs, KPC-15 showed more than two sites of amino acid changes (A119L and G146 K), and emerged to be homogenous with KPC-4 closly (Figures 1 and 2). The changes of the amino acids would influence the kinetic properties of the KPC enzymes. In spite of different values for the kms and kcats, the catalytic efficiency (kcat/km) of KPC-15 was higher than that of KPC-2 for all tested antibiotics (Table 3). These data implied that the effect of KPC-15 caused resistance to β-lactam antibiotics (including imipenem, meropenem, ceftazidime, cefotaxime, aztreonam and cefazolin) was higher than that of KPC-2. Moreover, an evolutionary transformation changed KPC from an efficient carbapenemase to its variants (KPC-15) with better ceftazidimase catalytic efficiency. Because of the multi-resistance to quinolones or to aminoglycosides antibiotics, in addition to carbapenemase, the strain Kp1241 might involve other genes, such as quinolone or aminoglycoside genes.

According to the literature, there is a distinct genetic environment with the chimera of several transposon-associated elements in China, such as Tn*3*, IS*Kpn8* and an IS*Kpn6*-like element on plasmid pKP048 [33]. With conjugation and gene mapping studies, we acquired three plasmids of different lengths. The *bla*_KPC-15_ variant is on a ca. 73-kb plasmid and carries I*SKpn8* and an IS*Kpn6*-like element determinant [24]. The transformant was also much less resistant than the original isolate, it was most likely due to the lack of any permeability lesion. The genetic environment of the *bla*_KPC-15_ gene in the plasmid showed a structural and context similarity to those characteristics found in plasmid pKP048 [33], except that a truncated *bla*_TEM-12_ gene and the *bla*_KPC-15_ variant were inserted and located between IS*Kpn8* and the novel *bla*_KPC-15_ variant [24]. The nucleotides upstream of the *bla*_KPC-15_ translational start codon were also shown in Figure 3. The start codon was indicated as G by the +1 residue, and the putative −10 promoter elements were shown as *gattaa* and labeled as −10 below. These data were consistent with a study by Yigit et al. [8]; however, no obvious −35 region was discovered in the promoter region, which was different from previous report [8]. Only a 233 bp sequence was discovered between the *bla*_TEM-12_ gene and the *bla*_KPC-15_ variant in this study (Figure 3).

## Conclusions

The study suggested that old antibiotics (polymyxin B and colistin) might play a role in therapy. The novel KPC-15 had more than two sites of amino acid changes (A119L and G146 K), and was homogenous with KPC-4 closly. The catalytic efficiency of KPC-15 causing resistance to β-lactam antibiotics was higher than that of KPC-2. Moreover, an evolutionary transformation changed KPC from an efficient carbapenemase to its variants (KPC-15) with better ceftazidimase catalytic efficiency.

## Supporting Information

Table S1 **The quantitative values of the initial rate versus substrate concentration (v_0_/[s]) for KPC-15 and KPC-2 enzymes.**
(DOC)
