# Supplementary material for: Phenotypic and Enzymatic Comparative Analysis of the KPC Variants, KPC-2 and Its Recently Discovered Variant KPC-15
Source: PLoS One. 2014 Oct 31;9(10):e111491. doi: 10.1371/journal.pone.0111491 (PMC4216079; doi:10.1371/journal.pone.0111491)
Supplement: Table S1 — The quantitative values of the initial rate versus substrate concentration (v0/[s]) for KPC-15 and KPC-2 enzymes. (DOC) [file pone.0111491.s001.doc]

Supplementary information:

Table S1. The quantitative values of the initial rate versus substrate concentration (v_0_/[s]) for KPC-15 and KPC-2 enzymes

| Substrate | KPC-15 |  | KPC-2 |
| --- | --- | --- | --- |
|  | v_0_/[s] (μM^-1^s^-1^) |  | v_0_/[s] (μM^-1^s^-1^) |
| Imipene | 0.0281 |  | 0.0095 |
| Meropenem | 0.0068 |  | 0.0003 |
| Ceftazidime | 0.0001 |  | 0 |
| Cefotaxime | 0.0092 |  | 0.0004 |
| Aztreonam | 0.0034 |  | 0.0014 |
| Cefazolin | 0.0562 |  | 0.0275 |
| Nitrocefin | 0.0915 |  | 0.0841 |
